# Technology Acceptance Among Low-Income Asian American Older Adults: Cross-Sectional Survey Analysis

**DOI:** 10.2196/52498

**Published:** 2024-11-22

**Authors:** Pauline DeLange Martinez, Daniel Tancredi, Misha Pavel, Lorena Garcia, Heather M Young

**Affiliations:** 1 Family Caregiving Institute Betty Irene Moore School of Nursing University of California, Davis Sacramento, CA United States; 2 Department of Pediatrics University of California, Davis Sacramento, CA United States; 3 Khoury College of Computer Science Northeastern University Boston, MA United States; 4 Department of Public Health Sciences University of California, Davis Sacramento, CA United States

**Keywords:** aged, older adults, Asian American, immigrant, vulnerable populations, internet, information and communications technology, ICT, digital divide, technology acceptance model, mobile phone

## Abstract

**Background:**

Studies show that the use of information and communications technologies (ICTs), including smartphones, tablets, computers, and the internet, varies by demographic factors such as age, gender, and educational attainment. However, the connections between ICT use and factors such as ethnicity and English proficiency, especially among Asian American older adults, remain less explored. The technology acceptance model (TAM) suggests that 2 key attitudinal factors, perceived usefulness (PU) and perceived ease of use (PEOU), influence technology acceptance. While the TAM has been adapted for older adults in China, Taiwan, Singapore, and Korea, it has not been tested among Asian American older adults, a population that is heterogeneous and experiences language barriers in the United States.

**Objective:**

This study aims to examine the relationships among demographics (age, gender, educational attainment, ethnicity, and English proficiency), PU, PEOU, and ICT use among low-income Asian American older adults. Two outcomes were examined: smartphone use and ICT use, each measured by years of experience and current frequency of use.

**Methods:**

This was a secondary data analysis from a cross-sectional baseline survey of the Lighthouse Project, which provided free broadband, ICT devices, and digital literacy training to residents living in 8 affordable senior housing communities across California. This analysis focused on Asian participants aged ≥62 years (N=392), specifically those of Korean, Chinese, Vietnamese, Filipino, and other Asian ethnicities (eg, Hmong and Japanese). Hypotheses were examined using descriptive statistics, correlation analysis, and hierarchical regression analysis.

**Results:**

Younger age, higher education, and greater English proficiency were positively associated with smartphone use (age: β=–.202; *P*<.001; education: β=.210; *P*<.001; and English proficiency: β=.124; *P*=.048) and ICT use (age: β=–.157; *P*=.002; education: β=.215; *P*<.001; and English proficiency: β=.152; *P*=.01). Male gender was positively associated with PEOU (β=.111; *P*=.047) but not with PU (β=–.031; *P*=.59), smartphone use (β=.023; *P*=.67), or ICT use (β=.078; *P*=.16). Ethnicity was a significant predictor of PU (*F*_4,333_=5.046; *P*<.001), PEOU (*F*_4,345_=4.299; *P*=.002), and ICT use (*F*_4,350_=3.177; *P*=.01), with Chinese participants reporting higher levels than Korean participants, who were the reference group (β=.143; *P*=.007). PU and PEOU were positively correlated with each other (*r*=0.139, 95% CI=0.037-0.237; *P*=.007), and both were significant predictors of smartphone use (PU: β=.158; *P*=.002 and PEOU: β=.166; *P*=.002) and ICT use (PU: β=.117; *P*=.02 and PEOU: β=0.22; *P*<.001), even when controlling for demographic variables.

**Conclusions:**

The findings support the use of the TAM among low-income Asian American older adults. In addition, ethnicity and English proficiency are significant predictors of smartphone and ICT use among this population. Future interventions should consider heterogeneity and language barriers of this population to increase technology acceptance and use.

## Introduction

### Background

Older adults increasingly use information and communications technologies (ICTs), including smartphones, tablets, computers, and the internet, to manage their health and finances, seek information, access services, and stay socially connected [1]. However, there are significant disparities in ICT use among older adults, particularly among those with low income [2]. According to the most recent study of intersectionality and technology use, only 46% of low-income adults aged ≥65 years in the United States used the internet as compared to 90% and 94% of mid-income and high-income older adults, respectively [3]. In addition, the ownership of smartphones, computers, and broadband subscriptions is lower among low-income older adults [4].

This digital divide is exacerbated among Asian American older adults, who experience higher rates of poverty (9.3%) as compared to the general older adult population in the United States (8.9%) [5]. In an analysis of the California Health Interview Survey (CHIS), the combination of Asian ethnicity and low income had an interactive, negative effect on ICT use [6]. In fact, Asian American older adults in the lowest income category were 14 times less likely to use the internet for health information compared to older non-Hispanic White individuals in the highest income category [6].

The COVID-19 pandemic highlighted the impact of limited digital access and skills among older adults. Compared to older adults with ICT access and proficiency, older adults who were not digitally connected faced health disparities, including social isolation [7,8], difficulties in accessing timely COVID-19–related information [9], and barriers to health care [10].

Asian American individuals are the fastest growing racial or ethnic group among adults aged ≥65 years in the United States [11,12]. In 2019, Asian Americans made up 4.6% of older adults in the United States, and they are projected to comprise approximately 8% of US older adults by 2060, at 7.9 million [13]. However, few studies have focused on technology use among Asian American older adults, despite indications that this population is among the least likely to use ICTs [6,14,15]. The 2011 National Health and Aging Trends Study indicated that Asian American older adults were less likely to send emails, SMS text messages, or conduct web-based tasks as compared to non-Hispanic White older adults [15]. Furthermore, a CHIS analysis between 2011 and 2016 found that internet use among non-Hispanic White older adults increased significantly from 66% to 73%; however, during this same period, internet use did not significantly change among Asian, Latinx, or Black older adults [14].

Asian Americans are frequently lumped together in research, but this population is heterogeneous, including >40 ethnicities, each with diverse cultural backgrounds, languages, and immigration histories [11,12,16]. Asian American ethnic groups vary significantly in income, educational attainment, English proficiency, and health status [17]. Few studies compare ICT use among Asian American ethnicities, though one exception is the study by Gordon and Hornbrook [18]. This analysis of a stratified, randomized survey of patients from a major health system in California revealed that Chinese older adults were significantly more likely to be using ICTs than Filipino older adults. While Chinese and non-Hispanic White older adults were equally likely to use the internet and access patient portals, Filipino older adults were less likely to own computers or smartphones, use the internet and email, or be willing and able to use digital technology to perform health-related tasks, including seeking health information [18]. Specifically, 84% of non-Hispanic White adults and 79% of Chinese adults aged 65 to 79 years reported being able to use the internet alone or with some help, as compared to only 53% of Filipino older adults. In addition, although 80% of non-Hispanic White older adults and Chinese older adults were able to send and receive email, only 60% of Filipino older adults were able to do so, even with help [18].

Limited English proficiency (LEP) is another characteristic that is negatively associated with ICT use. In a cross-sectional analysis of data from the 2011 National Health and Aging Trends Study, LEP was a significant, independent predictor negatively associated with using email and SMS text messaging, conducting web-based personal tasks, and seeking health information on the internet [15]. Furthermore, in an analysis of the CHIS, older adults with LEP were 53% less likely to report using the internet to seek health information as those who spoke English well or very well [6]. While these studies were not specific to Asian American older adults, LEP is a pertinent characteristic to consider when understanding technology use among Asian American older adults. According to the National Asian Pacific Center on Aging, >60% of Asian Americans aged ≥65 years have LEP, including >85% of Vietnamese older adults and >67% of Korean and Chinese older adults [11].

### Theoretical Framework

Over the past 4 decades, researchers have developed several models to describe the process of technology acceptance. The first models were based on the theory of planned behavior (TPB) by Ajzen [19], developed in 1980, which posits that one will not change their behavior until they have the psychological intent. The TPB suggests that 3 key factors, attitudes, subjective norms, and perceived behavioral control, influence intention. While the TPB is broadly about any behavior change, the technology acceptance model (TAM) proposed by Davis [20] adapted the TPB to describe factors that lead to technology use. The TAM proposes that perceived usefulness (PU) and perceived ease of use (PEOU) shape attitudes toward technology, which in turn shapes intention to use technology. PU refers to whether one perceives technology to be useful for what they hope to achieve, while PEOU refers to how much effort one expects to need to make to learn to use a new technology. PEOU has been operationalized to measure feelings of confusion, frustration, ease, predictability, intuitiveness of the system, or frequency of making mistakes [20].

While the TAM focused on understanding technology acceptance in the workplace [20], it has been adapted and tested with various subpopulations, in various geographies, and with a variety of technology applications. Later adaptations operationalized the variables using different measures, and they incorporated demographic factors as control variables in the model, including age, gender, and educational attainment [21,22].

Age has consistently been a significant, negative predictor of ICT use. According to 2017 Pew Research, 82% of adults aged 65 to 69 years in the United States reported using the internet; this number dropped to 75% for adults aged 70 to 74 years, 60% for adults aged 75 to 79 years, and 44% for adults aged ≥80 years [3]. Age is also negatively associated with smartphone ownership and home broadband subscription [3]. Baby boomers are more likely to have encountered ICTs in the workforce, leading to greater ICT acceptance and stronger digital skills [23-25]. In contrast, compared to baby boomers, adults aged ≥75 years experience greater technology anxiety, have lower technology proficiency, engage in less diverse web-based activities, and require more support to use ICTs [26-28].

Although gender is sometimes included as a control variable in the TAM [21,22], research examining the relationship between gender and ICT use has shown mixed results. While many studies found that older men are more likely to use the internet than older women [29-31], some recent studies have found either a shrinking gap between genders [29] or no difference [32]. Notably, a recent analysis of data from the Health and Retirement Study demonstrated that women aged ≥50 years were more likely to access the internet than men, although this difference decreased with age [33].

Educational attainment and ICT use are consistently, positively associated. In 2017, Americans aged ≥65 years who were college graduates were significantly more likely to use the internet, have a smartphone, and subscribe to home broadband (92%, 65%, and 82%, respectively), as compared to those who had a high school degree or less (49%, 27%, and 30%, respectively) [3]. In a cross-sectional analysis of a nationally representative sample of Medicare beneficiaries, education was one of the strongest predictors of internet use [34]. Compared to college graduates, high school graduates had 18% odds of using the internet, and those with less than a high school education had 8% odds of using the internet. In addition, those with a high school degree or less were likely to use the internet exclusively for emails and texting, while those with a college degree were more likely to engage in web-based shopping, banking, or health-related internet tasks [34].

While the TAM and its subsequent adaptations have been used to understand technology use among older adults living in China, Taiwan, Singapore, and Korea, the TAM has not been used to understand technology acceptance among Asian American older adults. Compared to Asian individuals living in their countries of origin, Asian American individuals are unique in their ethnic diversity. In addition, the majority of Asian American older adults in the United States experience language barriers [11]. While researchers have incorporated demographic variables such as age, gender, and educational attainment into the TAM, ethnicity and English proficiency are novel predictors that may increase the TAM’s ability to predict ICT use among Asian American older adults.

### Objectives

**Figure 1:**
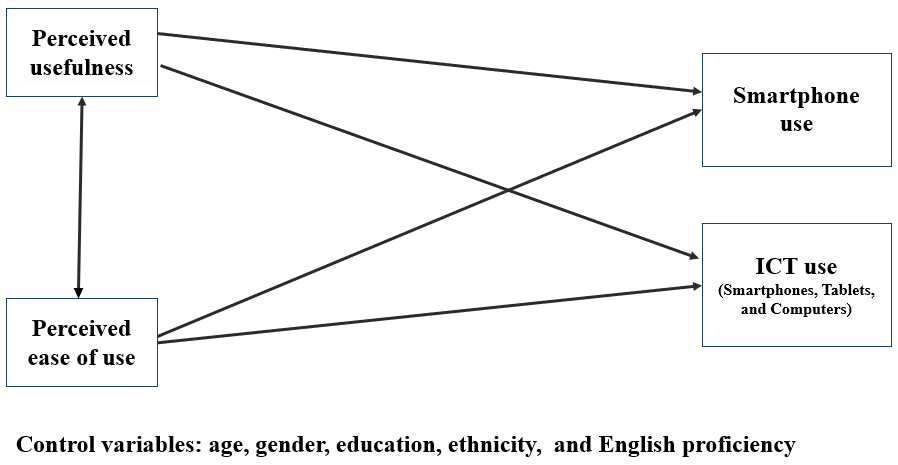
The technology acceptance model adapted for low-income Asian American older adults. ICT: information and communications technology.

This cross-sectional survey explored the relationships among age, gender, educational attainment, English proficiency, ethnicity, PU, PEOU, and ICT use among low-income Asian American older adults. This study examined 2 outcomes: smartphone use and ICT use. In addition, this study tested a simplified version of the TAM, as shown in Figure 1. In this adapted model, we removed the mediators from the original model (attitude and behavioral intention to use) [20]. Furthermore, this adaptation involved revising the TAM to shift the focus from workplace usefulness to perceptions of technology among older adults.

This study tested 4 hypotheses.

- Hypothesis 1: PEOU will be positively associated with PU.
- Hypothesis 2: age, gender, educational attainment, English proficiency, and ethnicity will be associated with PU and PEOU. We expect that age will be negatively associated with PU and PEOU; male gender, educational attainment, and English proficiency will be positively associated with PU and PEOU; and there will be heterogeneity in associations with PU and PEOU across Asian ethnicities.
- Hypothesis 3: age, male gender, educational attainment, English proficiency, and ethnicity will be associated with smartphone use and ICT use. We expect that age will be negatively associated with smartphone use and ICT use; male gender, educational attainment, and English proficiency will be positively associated with smartphone use and ICT use; and there will be heterogeneity in associations with smartphone use and ICT use across Asian ethnicities.
- Hypothesis 4: PU and PEOU will be positively associated with smartphone use and ICT use, even when accounting for age, gender, educational attainment, English proficiency, and ethnicity.

## Methods

### Ethical Considerations

This study was a secondary data analysis of the Lighthouse Project*.* On the basis of the HRP-210 Determination Request, the University of California, Davis, institutional review board determined that this research is exempt as it did not directly involve human participants and used deidentified secondary data (1938286-1).

### The Lighthouse Project

In partnership with 2 senior housing providers (Front Porch and Eskaton), the Lighthouse Project provided 8 affordable senior housing communities (open to residents aged >62 years) across California with high-speed broadband access, ICTs, and digital literacy training between July 2021 and July 2022 [35]. All residents (N=1050) were invited to participate. Recruitment occurred over a period of 1 to 6 months at each community during Wi-Fi installation. Onsite staff distributed flyers about the Lighthouse Project, set up an informational table in lobby areas or community rooms, and held social events where residents could test out a variety of ICT devices to inform device selection for the project and gather input. The Lighthouse Project participants completed surveys at entry and again at 30 and 90 days, received an ICT device, and were asked to attend a minimum of 1 of a series of digital literacy classes offered onsite at each community.

Hard copy surveys were distributed to residents’ apartments. Surveys were available in several languages; they were self-administered and collected by staff at each community. Staff were available to provide clarification and help with completing surveys, if needed. Most staff spoke English and at least 1 additional language, although on occasion, Google Translate or pocket translators were used to communicate with residents. Survey data were entered by affordable senior housing staff and verified by researchers from the University of California, Davis.

To address our study hypotheses, this study consisted of a secondary, cross-sectional analysis of a subset of Lighthouse Project baseline surveys. Participants were included if they reported being of Asian ethnicity and were aged ≥62 years. This age was selected based on the housing eligibility criteria. Participants were excluded if they had missing responses to any survey items that contributed to the 2 outcome variables (smartphone use and ICT use).

### Measures

#### Overview

In the Lighthouse Project, an evaluation expert and a gerontologist developed an evidence-informed survey that included demographic items and self-reported assessments of physical and emotional health, risk of depression, social isolation, and loneliness. It also featured technology-specific questions that examined attitudes toward ICTs, frequency and preferred use of devices, and available technology support. The survey was adapted for low-literacy levels, translated, and pilot-tested with affordable senior housing staff and residents across 2 communities representing 5 different languages (Chinese, Korean, Vietnamese, Spanish, and English). To minimize participant burden and increase accessibility, surveys were limited to 5 pages (front and back) and used large font with generous spacing.

#### Outcome Variables: Smartphone Use and ICT Use

In this study, smartphone use was operationalized as the standardized sum of 2 survey items. The first measured frequency of smartphone use (“How often do you use a smartphone [iPhone or Android]?”). Answer choices were coded as 0=never or I do not own, 1=once or less than once per week, 2=2-4 times per week, or 3=about once per day. The second item measured years of experience using ICTs (“How long have you been using technology, such as a computer, laptop, tablet or smartphone?”). Answer choices were coded as 0 (I have never used these), 1 (<1 year), 2 (1-2 years), or 3 (>2 years).

ICT use was operationalized as the sum of 4 survey items. Three separate survey items asked about frequency of use of computers, tablets, or smartphones (“How often do you use a desktop or laptop computer?” “How often do you use a tablet or iPad?” and “How often do you use a smartphone [iPhone or Android]?”). The fourth item inquired about years of experience using ICTs (“How long have you been using technology, such as a computer, laptop, tablet or smartphone?”).

Operationalization of outcome variables was assessed by 4 researchers with expertise in survey methodology with older adults, and data were reviewed by a biostatistician and a nurse researcher. When reviewing Predicted Probability plots, we observed that the residuals followed a normal distribution, closely aligning with the plots’ diagonal line. In addition, scatterplots of the residuals displayed no discernible pattern, with points evenly distributed both above and below 0 on the x-axis and to the left and right of 0 on the y-axis.

#### Attitudinal Factors: PU and PEOU

When Davis [20] developed the original TAM in the workplace, PU referred to usefulness in the work environment (eg, productivity and efficiency). As this study is focused on older adults, PU was adapted to encompass perceptions of technology as being useful for social connection, information seeking, and learning new skills [36]. Specifically, PU was operationalized as the standardized sum of 2 items, “Technology helps me be connected with family and friends” and “Technology helps me learn new information and skills.” Response categories for all statements ranged on a scale of 1 (strongly disagree) to 4 (strongly agree).

PEOU was operationalized as the standardized sum of 6 survey items combining 2 validated subscales. The Attitudes Toward Computers Questionnaire—Comfort Subscale [37] includes 4 items (“I feel comfortable with technology” [reverse scored], “Technology makes me nervous,” “I don’t feel confident about my ability to use technology,” and “Technology is confusing.”). The Senior Technology Acceptance Model—Technology Anxiety Subscale [22] includes 2 items (“I feel apprehensive about using technology” and “I hesitate to use technology for fear of making mistakes I cannot correct.”). Response categories for all statements ranged on a scale from 1 (strongly agree) to 4 (strongly disagree).

#### Demographic Factors

Demographic information included age (in years, centered at 80), gender (0=female, 1=male, and 2=other), and educational attainment (coded as –2=never attended school, –1=some high school, 0=completed high school or obtained a General Educational Development certificate, 1=some college, 2=college degree, or 3=graduate degree).

English proficiency was assessed by asking, “How well do you speak English?” Answer responses were coded as –1=not at all, 0=not well, 1=well, or 2=very well.

Five binary ethnicity variables were included in this study, including Korean, Chinese, Vietnamese, Filipino, or other Asian. Responses were mutually exclusive.

### Statistical Analysis

All data analysis was conducted using SPSS Statistics (version 28; IBM Corp). First, we computed descriptive statistics, including demographic characteristics, attitudes toward technology, and smartphone and ICT use. We assessed the distribution and internal consistency of the attitudinal variables, PU and PEOU, and the 2 composite outcome variables (smartphone use and ICT use).

Next, we used Pearson correlation analysis to examine relationships among all dependent and independent variables. To test hypothesis 1, we examined the unadjusted correlation between PU and PEOU.

To assess the other 3 hypotheses, hierarchical multiple regression was conducted using stepwise blocks [38]. To test hypothesis 2, outcome variables included PU and PEOU; to test hypotheses 3 and 4, outcome variables were smartphone use and ICT use. The sequence of inclusion of independent variables was established based on previous studies [22]. Demographic variables such as age, male gender, education level, English proficiency, and ethnicity (with Korean ethnicity as the reference group) were added in the first step (model 1). Next, attitudes toward technology (PU and PEOU) were added (model 2); finally, interaction terms were included in a forward stepwise regression (model 3). Interaction terms involving all pairwise combinations of demographic variables (except for ethnicity) with attitudinal variables (PU and PEOU) were also included. In addition, we evaluated the interaction of age and education, reasoning that female Asian Americans had systematic lower access to education in their native Asian country, and this may result in gender-specific adjusted associations of education with outcomes [39]. At each step, we assessed goodness of fit (adjusted *R*^2^), that is, the percentage of variability in the dependent variable that could be accounted for by the predictors. With each new set of terms, the change in *R*^2^ was calculated to quantify the change in the predictive power. The α level for testing significance was set to .05.

## Results

### Participation

In the Lighthouse Project, across the 8 affordable senior housing communities, 58% (609/1050) of the residents participated and 68% (414/609) of the Lighthouse participants were Asian. Two of the Lighthouse communities did not have Asian residents and were therefore excluded from this study.

Residents opted out of participating in the Lighthouse Project for several reasons, including fear or perceived burden of learning a new device or attending training classes; challenges related to vision, hearing, mobility, or cognitive decline; already having a device; or not finding the Lighthouse devices relevant.

After applying the inclusion and exclusion criteria, the final sample included 392 Asian residents aged ≥62 years, living in 6 affordable senior housing complexes located across California. Participants’ ages ranged from 62 to 97 years, with a mean of 79.1 (SD 6.95) years. Participant demographics are described in Table 1.

**Table 1:** Participant demographics (N=392)^a^.

Characteristic		Participants, n (%)	
**Age** **(y)**			
	62-74	105 (27.6)	
	75-84	192 (50)	
	>85	84 (22)	
**Gender**			
	Female	266 (68.2)	
	Male	124 (31.8)	
	Other	0 (0)	
**Ethnicity**			
	Chinese	73 (18.6)	
	Filipino	12 (3.1)	
	Korean	274 (69.9)	
	Other Asian (eg, Japanese or Hmong)	14 (3.6)	
	Vietnamese	19 (4.8)	
**English proficiency**			
	Very well	8 (2.1)	
	Well	63 (16.3)	
	Not well	198 (51.3)	
	Not at all	117 (30.3)	
**Educational attainment**			
	Never attended school	22 (5.9)	
	Some high school	133 (35.4)	
	Completed high school or obtained a General Educational Development certificate	76 (20.2)	
	Some college	62 (16.5)	
	College degree	60 (16)	
	Graduate degree	23 (6.1)	
**Years of experience using ICTs^b^**			
	>2	227 (57.9)	
	1-2	45 (11.5)	
	<1	30 (7.7)	
	I have never used these	90 (23)	
**Computer use**			
	About once/d	89 (22.7)	
	2-4 times/wk	30 (7.7)	
	Once or less than once/wk	27 (6.9)	
	Never	246 (62.8)	
**Tablet use**			
	About once/d	106 (27)	
	2-4 times/wk	35 (8.9)	
	Once or less than once/wk	20 (5.1)	
	Never	231 (58.9)	
**Smartphone use**			
	About once/d	243 (62)	
	2-4 times/wk	40 (10.2)	
	Once or less than once/wk	17 (4.3)	
	Never	92 (23.5)	
**Number of ICT device types used (computer, tablet, and smartphone)**			
	No devices	78 (19.9)	
	1 device	129 (32.9)	
	2 devices	77 (19.6)	
	3 devices	108 (27.6)	

^a^Missing data: 11 participants did not report their age, 2 participants did not report their gender, 6 participants did not report English proficiency, and 16 participants did not report their education level.

^b^ICT: information and communications technology.

### Internal Consistency of ICT Use and PEOU

We measured internal consistency of the items comprising the measures for ICT use and PEOU. The 4 items that made up ICT use had strong internal consistency and reliability, with a Cronbach α of 0.74. The 6 items that made up PEOU also had strong internal consistency and reliability, with a Cronbach α of 0.89.

### Correlation Analysis

Correlation analysis was used to examine relationships among demographic characteristics, attitudes toward technology, and smartphone and ICT use (Table 2).

**Table 2:** Correlation analysis (Pearson *r* and 2-tailed *P* value) among the research variables (N=392).

Variable		Smartphone use	ICT^a^ use	PU^b^	PEOU^c^	Age	Male gender	Educational attainment	English proficiency
**Smartphone use**									
	*r*	1	0.846^d^	0.234^d^	0.276^d^	–0.262^d^	0.127^d^	0.295^d^	0.256^d^
	*P* value	—^e^	<.001	<.001	<.001	<.001	.01	<.001	<.001
**ICT use**									
	*r*	0.846^d^	1	0.218^d^	0.327^d^	–0.225^d^	0.187^d^	0.321^d^	0.260^d^
	*P* value	<.001	—	<.001	<.001	<.001	<.001	<.001	<.001
**PU**									
	*r*	0.234^d^	0.218^d^	1	0.139^d^	–0.087	0.019	0.129^d^	0.064
	*P* value	<.001	<.001	—	.007	.10	.71	.01	.22
**PEOU**									
	*r*	0.276^d^	0.327^d^	0.139^d^	1	–0.141^d^	0.157^d^	0.186^d^	0.240^d^
	*P* value	<.001	<.001	.007	—	.006	.002	<.001	<.001
**Age** **(y)**									
	*r*	–0.262^d^	–0.225^d^	–0.087	–0.141^d^	1	–0.009	–0.106	–0.194^d^
	*P* value	<.001	<.001	.10	.006	—	.86	.04	<.001
**Male gender**									
	*r*	0.127^d^	0.187^d^	0.019	0.157^d^	–0.009	1	0.331^d^	0.189^d^
	*P* value	.01	<.001	.71	.002	.86	—	<.001	<.001
**Educational attainment**									
	*r*	0.295^d^	0.321^d^	0.129^d^	0.186^d^	–0.106	0.331^d^	1	0.403^d^
	*P* value	<.001	<.001	.01	<.001	.04	<.001	—	<.001
**English proficiency**									
	*r*	0.256^d^	0.260^d^	0.064	0.240^d^	–0.194^d^	0.189^d^	0.403^d^	1
	*P* value	<.001	<.001	.22	<.001	<.001	<.001	<.001	—
**Korean**									
	*r*	0.002	–.095	–0.203^d^	–.131^d^	0.003	0.014	–0.015	0.020
	*P* value	.97	.06	<.001	.01	.96	.78	.77	.70
**Chinese**									
	*r*	–0.045	.051	0.171^d^	0.058	0.102	0.030	–0.048	–0.280^d^
	*P* value	.38	.31	.001	.25	.047	.56	.36	<.001
**Vietnamese**									
	*r*	0.066	.100	0.049	–0.014	–0.113	–0.001	0.002	0.079
	*P* value	.19	.049	.35	.79	.03	.98	.97	.12
**Filipino**									
	*r*	0.109	.104	0.091	0.220^d^	–0.077	–0.090	0.172^d^	0.329^d^
	*P* value	.03	.04	.08	<.001	.13	.08	.001	<.001
**Other Asian**									
	*r*	–0.088	–.084	–0.002	0.011	–0.012	–0.013	–0.028	0.142
	*P* value	.08	.10	.97	.83	.81	.79	.59	.005

^a^ICT: information and communications technology.

^b^PU: perceived usefulness.

^c^PEOU: perceived ease of use.

^d^The correlation is significant at a significance level of .01 (2-tailed).

^e^Not applicable.

PU and PEOU were positively, significantly associated with one another (*r*=0.139, 95% CI 0.037 to –0.237; *P*=.007). Hence, hypothesis 1 (PEOU will be positively associated with PU) was supported; we reject the null hypothesis.

### Regression Analysis

Results of hierarchical regression are presented in Tables 3 and 4. Tolerance values of all independent variables were >0.01, and variance inflation factor values were <5, indicating lack of multicollinearity [40].

**Table 3:** Results of hierarchical regression: perceived usefulness (PU) and perceived ease of use (PEOU) as dependent variables (N=392)^a^.

Model 1: independent variables			PU				PEOU		
			β		*P* value		β		*P* value
Age			–.084		.12		–.099		.06
Male gender			–.031		.59		.111		.047
Education			.121		.047		.045		.44
English proficiency			.039		.55		.148		.02
**Ethnicity**									
	Chinese	.231		<.001		.127		.02	
	Filipino	.074		.20		.179		.001	
	Korean	Reference		Reference		Reference		Reference	
	Other Asian	–.021		.70		.001		.99	
	Vietnamese	.068		.20		–.019		.71	
Adjusted *R*^2^			.058		—		.098		—

^a^β values are standardized regression coefficients.

**Table 4:** Results of hierarchical regression: smartphone and information and communications technology (ICT) use as dependent variables (N=392)^a^.

Model and independent variables				Smartphone use				ICT use		
				β		*P* value		β		*P* value
**1**										
	Age		–.202		<.001		–.157		.002	
	Male gender		.023		.67		.078		.16	
	Education		.210		<.001		.215		<.001	
	English proficiency		.124		.048		.152		.01	
	**Ethnicity**									
		Chinese	.016		.76		.143		.007	
		Filipino	.017		.75		.019		.72	
		Korean	Reference		Reference		Reference		Reference	
		Other Asian	–.124		.02		–.089		.08	
		Vietnamese	.025		.63		.077		.13	
	Adjusted *R*^2^		.137		—		.155		—	
**2**										
	Age		–.171		.001		–.124		.01	
	Male gender		.010		.86		.057		.29	
	Education		.184		.001		.191		.001	
	English proficiency		.096		.12		.118		.05	
	**Ethnicity**									
		Chinese	–.041		.44		.089		.09	
		Filipino	–.025		.65		–.030		.58	
		Korean	Reference		Reference		Reference		Reference	
		Other Asian	–.118		.02		–.083		.09	
		Vietnamese	.018		.73		.074		.13	
	PU		.158		.002		.117		.02	
	PEOU		.166		.002		.221		<.001	
	Adjusted *R*^2^		.184		—		.211		—	
	Δ*R*^2^		.047		<.001		.056		<.001	
**3**								—		—
	Age		–.166		.001		—^b^		—^b^			
	Male gender		.009		.86		—^b^		—^b^			
	Education		.168		.004		—^b^		—^b^			
	English proficiency		.111		.07		—^b^		—^b^			
	**Ethnicity**									
		Chinese	–.057		.29		—^b^		—^b^	
		Filipino	.019		.74		—^b^		—^b^	
		Korean	Reference		Reference		—^b^		—^b^	
		Other Asian	–.110		.03		—^b^		—^b^	
		Vietnamese	.006		.91		—^b^		—^b^	
	PU		.183		.001		—^b^		—^b^	
	PEOU		.157		.003		—^b^		—^b^	
	English proficiency×PEOU		–.120		.03		—^b^		—^b^	
	Adjusted *R*^2^		.193		—		—^b^		—^b^	
	Δ*R*^2^		.009		.03		—^b^		—^b^	

^a^β values are standardized regression coefficients.

^b^No interaction terms were kept in a stepwise regression.

### Testing Hypothesis 2 (Model 1: PU and PEOU as Outcome Variables)

As expected, educational attainment was positively, significantly associated with PU when controlling for age, gender, English proficiency, and ethnicity. In addition, we found heterogeneity in associations with PU across Asian ethnicities; the omnibus *F* test for assessing the adjusted association of the 5-level ethnicity classification with PU was statistically significant (*F*_4,333_=5.046; *P*<.001). Specifically, Chinese ethnicity was significantly, positively associated with PU as compared to the reference level (Korean ethnicity). However, contrary to expectation, age, gender, and English proficiency were not significant predictors of PU.

Male gender and English proficiency were each significant, positive predictors of PEOU when controlling for age, educational attainment, and ethnicity. In addition, we found heterogeneity in association with PEOU across Asian ethnicities; the omnibus *F* test for assessing the adjusted association of the 5-level ethnicity classification with PEOU was statistically significant (*F*_4,345_=4.299; *P*=.002). Contrary to expectation, age and educational attainment were not significant predictors of PEOU. Therefore, hypothesis 2 (age, gender, educational attainment, English proficiency, and ethnicity will be associated with PU and PEOU) was partially supported.

### Testing Hypothesis 3 (Model 1: Smartphone Use and ICT Use as Outcome Variables)

As expected, age was significantly, negatively associated with smartphone use, and educational attainment and English proficiency were significantly, positively associated with smartphone use, when controlling for other demographic variables. Contrary to hypothesis 3, male gender was not a significant predictor of smartphone use. In addition, ethnicity was not a significant predictor of smartphone use; the omnibus *F* test for assessing the adjusted association of the 5-level ethnicity classification with smartphone use was not statistically significant (*F*_4,340_=1.619; *P*=.17). Nevertheless, indicator variables for ethnicity were still included in the overall model (with Koreans as the reference category), and regression analysis revealed that participants of “other Asian” ethnicity were significantly less likely than Koreans to be using smartphones.

Likewise, age was significantly, negatively associated with ICT use, and educational attainment and English proficiency were significantly, positively associated with ICT use, when controlling for other demographic variables. While ethnicity was a significant predictor of ICT use when controlling for age, gender, education, and English proficiency, significance was lost when we added PU and PEOU to the model. The omnibus *F* test for assessing the adjusted association of the 5-level ethnicity classification in the final model (Table 4: model 3) was *F*_4,340_=2.087; *P*=.08. Finally, contrary to hypothesis 3, male gender was not a significant predictor of ICT use. Therefore, overall, hypothesis 3 (age, male gender, educational attainment, English proficiency, and ethnicity will be associated with smartphone use and ICT use) was partially supported.

### Testing Hypothesis 4 (Models 2 and 3: Smartphone Use and ICT Use as Outcome Variables)

Adding PU and PEOU to the model significantly strengthened the predictive power of smartphone use and ICT use. Hypothesis 4 was fully supported; PU and PEOU were significant, positive predictors of smartphone use and ICT use, when accounting for age, gender, educational attainment, English proficiency, and ethnicity.

There was a significant interaction between PEOU and English proficiency in the model predicting smartphone use. None of the interaction terms tested were significant in the model predicting ICT use.

## Discussion

This was the first study to test the TAM [20] among low-income Asian American older adults. We sought to understand what demographic factors predict smartphone use and ICT use in this population.

### Smartphone Use

Approximately three-quarters of participants (300/392, 76.5%) reported that they use a smartphone, with most of these (283/300, 94.3%) reporting that they use a smartphone at least twice per week. These findings are high compared to a nationally representative study from 2022 that reported that 61% of Americans aged ≥65 years (of all incomes) owned a smartphone [41]. We would expect our sample to have lower smartphone ownership due to their income [42].

However, it is also possible that participants in our study confused smartphones and regular mobile phones, leading to overreporting smartphone use. According to 2017 Pew Research, 80% of Americans aged ≥65 years owned a cellphone of any kind (including smartphones and nonsmartphones) [3], and similarly, Gordon and Hornbrook [18] found that 81% of patients aged 65 to 79 years from a large health plan in California reported owning a cell phone of any kind. These percentages are similar to the prevalence of smartphone use that we found in our study (300/392, 76.5%). While our survey defined smartphones as “an iPhone or Android,” this verbiage may not have been sufficient. During the Lighthouse Project (the parent study for this analysis), affordable senior housing residents expressed confusion about terminology such as “tablet” and “Wi-Fi.” As older adults did not grow up with the technology available today, it is particularly important to use clear and explicit language to avoid confusion and inferences [43]. In addition, translating technology terms to other languages may contribute to further misunderstandings.

### Tablet Use

Tablet ownership in our sample was similar to the national average, with 41.1% (161/392) of the participants reporting that they use a tablet and 87.3% (141/161) of the tablet users saying that they use their tablet at least twice per week. In comparison, in 2021, in total 44% of Americans aged ≥65 years reported owning a tablet [41]. However, we expected to see lower tablet ownership in our study population because all participants had low income, while the previous study included individuals from all income levels [42].

### Computer Use

A little more than one-third of the participants (146/392, 37.2%) reported that they use a computer (desktop or laptop), with 81.6% (119/146) of these reporting that they use a computer at least twice per week. In comparison, in the study by Gordon and Hornbrook [18] including patients aged 65 to 79 years from a large health system in California, 81.5% reported having access to a desktop, laptop, or notebook computer.

While computer use appears to be low in our population, these studies are difficult to compare as our study asked about “use,” while the previous study focused on “access.” This is a common challenge across the research literature related to technology acceptance; researchers use a variety of measurement constructs, and outcomes can vary widely, including “intention to use,” “access,” and “actual use" [26,44]. Furthermore, most studies depend on self-reported data, which limits their validity.

### Demographic Associations With PU, PEOU, Smartphone Use, and ICT Use

#### Age and Education

Our regression analysis revealed that among low-income Asian American adults aged ≥62 years, younger age and greater educational attainment are independently, positively associated with smartphone use and ICT use. This is consistent with studies conducted with older adults living in the United States [3,27,45], China [46], and Korea [47]. Although these other studies evaluated various outcomes, they consistently found positive relationships between younger age, higher education, and constructs related to ICT use. For example, in a nationally representative survey of Americans aged ≥65 years, there were independent, positive associations between younger age, educational attainment, smartphone ownership, and internet use [3]. In a cross-sectional survey of New Englanders aged ≥65 years, age and educational attainment predicted ICT use [45]. Another survey study of 500 Americans aged ≥60 years found significant associations among age, education, ICT access, and internet skills [27]. In a study in China, age and education were significant factors predicting smartphone acceptance among older adults [46]. Finally, in a study in Korea, educational attainment was a significant predictor of technology acceptance among older adults [47].

Although age was a predictor of smartphone and ICT use, in our study, it did not emerge as a significant predictor of PU or PEOU when controlling for other variables. We also found that education was positively, significantly associated with PU, but not PEOU, when accounting for age, gender, educational attainment, English proficiency, and ethnicity. These findings are consistent with those of Mitzner et al [48], who also found that among low-income older adults, age was not significantly associated with PU or PEOU, and education was not associated with PEOU. In contrast, other studies that engaged a broader range of age groups (not only older adults) found significant relationships between age and PU, PEOU, computer anxiety, and computer self-efficacy [49,50].

#### Gender

We did not find significant associations between gender and smartphone use or ICT use. These findings are consistent with several other studies [29,32,45,48,51,52]. Interestingly, male gender was a significant, positive predictor of PEOU, although it was not associated with PU. In a systematic review focused on gender differences in technology use, men were more technologically adept than women [53]. Other studies suggest that women and men use ICTs in different ways, particularly when it comes to maintaining social relationships [53,54]. Some researchers explored the impact of the cultural construct of masculinity in Asian cultures on technology acceptance [55,56]. According to Hofstede [57] and Hofstede and Bond [58], masculinity-femininity refers to the preference for ambition or material success. Masculine cultures stress greater gender roles, and this may lead Asian men to invest more time in learning to use technology as compared to Asian women, possibly increasing men’s PEOU [55,56].

#### English Proficiency

English proficiency had significant adjusted associations with smartphone use and ICT use, as well as with the mediator PEOU, but not PU. Further investigation showed that English proficiency remained associated with ICT use when adjusting for the mediators PU and PEOU. For the smartphone use outcome, English proficiency modified the association of the mediator PEOU. These findings suggest that participants with LEP feel less comfortable using ICTs, and this in turn may lead to lower smartphone and ICT use. Our findings align with those of others who found that LEP was a significant, independent predictor negatively associated with using email and SMS text messaging, conducting web-based personal tasks, and seeking health information on the internet in a nationally representative sample of older adults [15]. It is important to note that, in our study, participants who were Filipino or in the “other Asian” category were more likely to be proficient in English. This is expected for the Filipino population, as English is one of the official languages in the Philippines.

Language barriers can hinder the ability to access relevant information on the internet, navigate user interfaces, and communicate effectively. Many websites, apps, and user interfaces are limited in non-English languages [59]. Most online content is in English, and other Asian languages, such as Vietnamese, are significantly less represented on the internet [60]. Though there are an estimated 6000 languages used around the globe today, Google Search is only available in 130 different languages [59]. Availability of mobile apps also varies by language. The iOS App Store offers mobile apps in only 40 languages, and many popular apps are only offered in a handful of languages [61]. In addition, popular voice user interfaces, such as Google Voice and Alexa, operate in limited languages; for example, Google Assistant does not function in Cantonese [62]. Individuals who speak English as a second language face multiple challenges when learning to use Google Home smart speakers; they struggled with using structured voice commands, choosing the “right” words to activate the device, and pronouncing instructions in a manner that the Google voice interface can recognize [63].

Other studies have focused on LEP in the context of health-related ICT use. Health portals, websites, mobile health apps, and digital health interventions are often challenging for those with LEP to use [64-66]. In addition, Californian older adults with LEP were 53% less likely to report using the internet to seek health information compared to those who indicated they spoke English well or very well [6].

#### Ethnicity

Ethnicity was a significant predictor of PU, PEOU, and ICT use, although it was not a significant predictor of smartphone use. Specifically, with Korean participants as the reference group, Chinese ethnicity was positively associated with PU, PEOU, and ICT use when adjusting for age, gender, educational attainment, and English proficiency. In addition, Filipino ethnicity was associated with greater PEOU compared to Korean ethnicity.

Few previous studies have compared technology acceptance across Asian ethnicities. One exception was the finding that Chinese older adults were significantly more likely to use ICTs than Filipino older adults in California [18]. Another study found that low-income older adults living in Korea had greater comfort and use of ICTs as compared to low-income older adults living in the United States [67]. In Korea, internet is a prerequisite for all types of daily activities; broadband is cheap and accessible; and Korean apps (such as Kakao Talk) are ubiquitous, even among low-income older adults [67]. The year in which someone immigrated from Korea to the United States may impact their technology acceptance.

Yoon et al [68] suggested that acculturation also plays a role in technology acceptance. In a cross-sectional survey analysis of Korean Americans aged ≥60 years, acculturation was a significant predictor of computer use and computer anxiety. Acculturation was defined as the degree to which a person from another culture learned the new language, customs, and behaviors of the host culture [68].

### Testing the TAM

Overall, our regression analysis supported the TAM [20]. We found that PU and PEOU were positively, significantly associated with one another, and PU and PEOU were significantly, positively associated with our 2 outcome variables—smartphone use and ICT use. This remained true when accounting for age, gender, educational attainment, English proficiency, and ethnicity. Our findings align with subsequent TAM adaptations, such as the unified theory of acceptance and use of technology [21], TAM2 [69], TAM3 [70], and unified theory of acceptance and use of technology 2 [71], as well as studies conducted among older adults in China [71,72], Taiwan [73], and Korea [47,74,75].

In contrast, Chen and Chan [22] found that PU and PEOU were not significant predictors of technology use among a sample of Hong Kong Chinese adults aged ≥55 years. Instead, health and social factors were better predictors of technology use [22]. Because of their findings, Chen and Chan [22] proposed the senior technology acceptance model, which added health conditions, cognitive ability, physical function, and social relationships as independent variables predicting technology acceptance. While we did not account for health and social factors in this study, in future studies, we plan to explore how health and social factors impact PU, PEOU, smartphone use, and ICT use among Asian American older adults.

### Limitations

Our study had limitations. We conducted a secondary analysis of data from the Lighthouse Project, using surveys that were administered before the design of this study. While we operationalized variables to match concepts from the original TAM, our measures differ from other TAM studies, making it difficult to compare findings. This is a common problem across research studies examining technology acceptance among older adults [26,44].

The measures used in this study were evidence based and translated from English to Korean, Chinese, and Vietnamese, although the translated items were not validated in all these languages. Vocabulary used and subsequent translation may have impacted data validity. In addition, study participants reported low educational attainment, and qualitatively, staff shared that some residents had low literacy.

Survey results were further limited by response bias and self-report. According to Deng et al [51], older adults tend to overestimate their smartphone use. Future studies seeking to understand ICT acceptance among older adults would benefit from collecting objective ICT use data.

This study engaged a convenience sample; our sample was not representative of Asians across California or the United States. Most of our participants were Korean, and the sample sizes representing Vietnamese and Filipino ethnicities were small. In addition, although surveys were collected across 6 communities, 70.7% (277/392) of our surveys were collected from residents living in 2 communities, which house primarily Korean residents and are both located in Koreatown, Los Angeles. Residents in these communities may experience less linguistic isolation than the monolingual Asian residents in the other Lighthouse communities. Finally, many other factors potentially impact ICT use among Asian American older adults, such as health status, access to Wi-Fi, availability of devices, digital literacy training, and technology support.

### Conclusions

This study contributed to our insights of factors that influence ICT acceptance and use among community-dwelling Asian American older adults, specifically those of Korean, Chinese, Vietnamese, and Filipino ethnicities (N=392). By increasing the understanding of the associations among age, gender, educational attainment, English proficiency, ethnicity, attitudes toward technology, and ICT use, our findings can inform digital interventions aimed at addressing disparities among Asian American older adults. For example, our study suggests that older age, lower educational attainment, and LEP are negatively associated with smartphone and ICT use. To minimize disparities, interventions could be targeted toward Asian older adults with these characteristics.

Furthermore, PU and PEOU are potentially modifiable factors that can be addressed through group-based or one-on-one digital literacy training and support to new learners [76-79]. The technology industry could address access barriers by creating tailored user interfaces including voice user interfaces that minimize the burden for individuals with low literacy [80,81]. Our study found that female gender, LEP, and Korean ethnicity were each independently, negatively associated with PEOU, and limited educational attainment and Korean ethnicity were negatively associated with PU. Future research could explore how interventions influence attitudes such as PU or PEOU in this subpopulation to increase ICT acceptance and use.

There is a need for validated measures of ICT acceptance and use targeted toward older adults; these measures should be brief to minimize participant burden, continuously updated to keep pace with technology innovation, accessible for those with low literacy, and validated in multiple languages to allow greater understanding of technology acceptance among older adults with LEP.

Future studies should involve larger, more representative samples of older Asian Americans, including diverse ethnic groups. As highlighted, Asian Americans comprise >40 ethnicities, each with unique cultures, immigration backgrounds, and languages [11,12,16], and our findings demonstrate significant variations in technology acceptance and use across these groups.

The impact of English proficiency on ICT use warrants deeper exploration to identify strategies for enhancing equity and access for individuals with primary languages other than English. Further research is needed to understand the interplay of age, education, English proficiency, smartphone use, and ICT adoption, particularly considering the distinct challenges this combination poses for older adults using technology.

Interventional studies are needed to explore effective strategies to overcome resistance to technology, support novice users in adopting new devices, and broaden the diversity of technology use among comfortable users beyond basic functions. Qualitative and ethnographic studies could further elucidate the barriers and facilitators of technology adoption. Finally, future research should explore how other factors, such as health and social factors, influence ICT acceptance and use among Asian American older adults.

## Abbreviations

CHIS: California Health Interview Survey
ICT: information and communications technology
LEP: limited English proficiency
PEOU: perceived ease of use
PU: perceived usefulness
TAM: technology acceptance model
TPB: theory of planned behavior

## Data Availability

The datasets generated or analyzed during this study are available from the corresponding author on reasonable request.
